# Supplementary material for: Recent quantitative research on determinants of health in high income countries: A scoping review
Source: PLoS One. 2020 Sep 17;15(9):e0239031. doi: 10.1371/journal.pone.0239031 (PMC7498048; doi:10.1371/journal.pone.0239031)
Supplement: S1 Appendix — (DOCX) [file pone.0239031.s002.docx]

# S1 Appendix

| **Database** | **Search query** | **Yield as of the 16^th^ of July, 2019** |
| --- | --- | --- |
| PubMed | (“infant survival” OR “mortality” OR “DALY” OR “disability-adjusted life year” OR “disability-adjusted life years” OR “disability adjusted life year ” OR “disability adjusted life years” OR “QALY” OR “quality-adjusted life year” OR “quality-adjusted life years” OR “quality adjusted life year” OR “quality adjusted life years” OR “life expectancy” OR “life expectancies” OR “life-expectancy” OR “longevity” "HALE" OR "PYLL" OR "potential years of life lost" OR “population health” OR “public health” OR “health outcomes” OR “health status” OR "health indicators" OR "health production" OR "health determinants" OR "determinants of health" OR "determinants of healthcare" OR "determinants of health-care") AND (“cross-country” OR “cross-nation” OR “cross-national” OR “countries” OR “across nations” OR “multicountry” OR “multi-country” OR “Europe” OR “EU” OR “European” OR “OECD” OR “Organization for Economic Cooperation and Development” OR “Organization for Economic Co-operation and Development” OR “Organisation for Economic Cooperation and Development” OR “Organisation for Economic Co-operation and Development” OR "Africa" OR "African" OR "America" OR "Americas" OR "American" OR "South-East Asia" OR "South East Asia" OR "Eastern Mediterranean" OR "Western Pacific") | 964 |
| Web of Science | (“infant survival” OR mortality OR DALY OR “disability-adjusted life year$” OR QALY OR “quality-adjusted life year$” OR life-expectanc* OR longevity OR HALE OR PYLL OR "potential years of life lost" OR “population health” OR “public health” OR “health outcome$” OR “health status” OR "health indicator$" OR "health production" OR health determinant$ OR healthcare determinant$ OR health-care determinant$) AND (cross-country OR cross-nation* OR countries OR across nations OR multicountry OR multi-country OR Europe OR EU OR European OR OECD OR “Organi?ation for Economic Cooperation and Development” OR “Organi?ation for Economic Co-operation and Development” OR Africa OR African OR America$ OR "South-East Asia" OR "Eastern Mediterranean" OR "Western Pacific") | 3619 |
