# Supplementary material for: Recent quantitative research on determinants of health in high income countries: A scoping review
Source: PLoS One. 2020 Sep 17;15(9):e0239031. doi: 10.1371/journal.pone.0239031 (PMC7498048; doi:10.1371/journal.pone.0239031)
Supplement: S2 Appendix — (DOCX) [file pone.0239031.s003.docx]

# S2 Appendix

Based on the predictor variables utilized across our selected studies, a data matrix to reflect “proximities” among them was devised. All predictors, regardless of whether they had been considered a main covariate, control, confounder, moderator or mediator, were taken into account. In the few instances where a predictor was, in fact, one of our outcomes of interest (1-3), that predictor was disregarded. In order to prevent variable clutter, we treated groups of predictors that operationalized or measured the same general concept differently as one unit. S1 Table shows the groupings we made in order to reduce the total number of variables to 88. The actual values in the data matrix corresponded to the number of times a particular pair of predictors had been considered simultaneously, in the same analysis, regardless of whether that was the main analysis of the study, a sensitivity or a supplementary one. The matrix was symmetric with a 0-diagonal and off-diagonal values ranging from 0 to 16.

**S2 Table. Determinants grouping for the purposes of multidimensional scaling**

**variable groupings (used in…)**

**age**

population age distribution (<15, 15-64, 65+) (3, 4)

%population 65yrs and older (3, 5-10)

median age of the pop (3)

aging index (3)

index of economic dependence of young people (3)

index of economic dependence of old people (3)

**alcohol**

alcohol consumption (5, 11-13)

spirits consumption (11)

recorded & unrecorded per capita (15+) alcohol consumption (in litres of pure alcohol) (14)

**birth rate (BR)**

gross birth rate (3)

live births (3)

**corruption**

Corruption Perceptions Index (15, 16)

corruption index (indicator of institutional development) (1)

**culture**

Inglehart scales: self-expression (and variants post-materialism & autonomy/socio-liberalism), secular-rational (and variants religiosity & normative-religious) (11)

Hofstede indices: power distance, individualism, uncertainty avoidance, masculinity, long-term orientation, indulgence (11)

Schwarts orientations: affective autonomy, intellectual autonomy, embeddedness, egalitarianism, hierarchy, harmony, mastery (11)

**democracy**

revised Polity2 index (17, 18)

current democracy (17)

cumulative years of democracy (17)

**drugs**

%pop 15-64yo consuming opiates (14)

injected drugs prevalence 15-64yo (14)

**education (EDU)**

%population over 25yo with no education (19)

%population over 25yo with post-secondary diploma (19)

average years of schooling (13, 17, 20)

primary school enrollment Blazquez-Fernandez’18

gross enrollment ratio in secondary school (15)

education expenditure (5)

%population with tertiary education (5, 10)

%population with upper secondary education (5)

%population with primary education (5)

average levels of education (11)

ratio of female to male primary, secondly and tertiary school enrollment (21)

attained tertiary education degree among 25–64 yo's (1, 22)

%population 25-64yo with tertiary education (23)

%population completed at least lower secondary education (24)

proportion of ppl 25-64yo w/ only lower secondary edu (25)

share of pop w/ basic secondary edu (26)

**freedom**

economic freedom (15, 17, 20)

societal freedom (15)

freedom of the press (15)

**gender**

%females (12)

%males (8)

**gross domestic product (GDP)**

GDP per capita (1, 2, 4-13, 15-20, 22-25, 27-41)

gross national income per capita (GNI) (21, 42)

regional income (43)

gross added value (3)

**health(care) expenditure (HE)**

total health-care expenditure (13-15, 44)

public health expenditure (2, 24)

government health-care spending (6)

out-of-pocket expenditure (6, 9)

private health expenditure (6)

publicly funded health-care (1)

public health expenditure (9, 26, 45)

private health expenditure (45)

government health expenditure (8)

government health-care expenditure (4)

HC effort (public health care spending as a percent of GDP) (10)

pub.sector share (PSS, public HC spending as % of total) (10)

**income**

compensation of employees pre inhabitant (32)

household income (3)

**income inequality (inc.ineq)**

Gini index of income inequality (1, 4, 10, 20, 22, 24, 31, 33, 35, 36)

income inequality (the ratio between the top and bottom 20%) (46, 47)

Gini coefficient of equalised disposable income (44)

gross earnings ratio (earnings of those in the 1st decile of the earnings distribution to that of those in the 9th decile) (35)

S80/S20 income quintile ratio (44)

Kaitz index (8)

**leave**

job-protected paid leave (37)

other leave (37)

job-protected paid parental leave (39)

**maternal age (M-age)**

average age of females at first birth (2, 24)

teenage pregnancy (% of all live births) (11)

%live births to mothers 35yo or older (11)

%live births to mothers younger than 20y (11, 24)

mean maternal age at birth (3)

**migration**

gross rate of natural movement of pop (3)

natural movement of pop (3)

gross migration rate (3)

net migration (rate of population change not accounted for by births & deaths) (10)

**poverty**

child income poverty (<50% of median) (22)

neighborhood socio-economic deprivation (Caranci index) (48)

%population at risk of poverty, severely materially deprived or living in households with very low work intensity (24)

European Deprivation Index (EDI) (49)

social exclusion (proportion of ppl at risk of poverty or soc.exclusion) (25)

**smoking**

%daily smokers (5, 11, 12)

%daily male smokers (5)

%daily female smokers (5, 22)

tobacco consumption per adult (13, 50)

cigarette consumption (>15yo, ave per day) (10)

smoking prevalence among males (14)

**social expenditure (soc.E)**

social expenditure per capita (in the areas of old age, survivors, incapacity-related benefits, family, active-labor market programs, unemployment, housing, other areas including noncategorical cash benefits to low-income households, food subsidies) (5, 9)

public social spending (30)

public expenditure (direct in-kind provision of goods & services, cash benefits, and tax breaks with social purposes) (12)

social protection spending (43)

social spending (1)

public spending on family benefits in cash, services and tax measures (22)

expenditures on family cash allowances (37)

expenditures on maternity & parental leave (37)

expenditures on family services (37)

public spending (8)

family benefits (39)

social spending (both cash and in-kind, on 6 domains: education, family, unemployment, incapacity, old age, active labor market programs) (4)

**trust**

most people can be trusted (%pop) (22)

trust in society (%pop) (16)

**unemployment rate (UR)**

UR (2, 3, 6, 10, 13, 19, 23, 24, 30, 31, 38, 43, 51)

long-term UR (3, 25)

employment-to-population ratio (51)

economically active population (3)

employment (3)

employment rate (3)

economic activity rate (3)

**vaccination**

vaccination rate (%children younger than 2yo vaccinated for DPT) (2)

immunization rates for measles by age 1 (13, 37)

immunization rates for DPT by age 1 (37)

**veg-fr**

fruit and vegetable consumption (kg pc per year) (14)

vegetable consumption pc (13)

Our goal was to obtain a spatial representation of the position of each predictor in relation to the others, based on how often joint effects on health had been investigated. The multidimensional scaling (MDS) technique was used for the purpose. First, the proximity data matrix was converted into a distance one, subtracting all non-diagonal values from 17, the value 17 signifying the greatest possible distance (i.e., the pair was never considered together) and the value 1 – the smallest observed distance (variables from the GDP and the HE groups were examined in combination 16 times in total). Both metric (absolute, ratio, and interval) and non-metric (ordinal) MDS methods were applied. Uni-dimensional up to 15-dimensional solutions were examined. The phenomenon of degeneracy was evident in all models though, rendering the acceptable badness-of-fit criterion of high-dimensional solutions unreliable (52).

Degeneracy typically occurs when points are gathered closely around a small number of locations, possibly due to some natural clustering in the data themselves. Indeed, this was to be expected with our data matrix as it contains only 17 unique values in the 3828 cells of either the lower or upper triangular half. Thus, many predictor variables, especially the ones with minimal or no “connections” with the others, would appear functionally identical from an MDS perspective. Even though this problem was somewhat less severe with high-dimensional solutions using the absolute and ratio approaches, for simple visualization purposes we chose to focus on a 2-dimensional non-metric model. The non-metric approach, also called ordinal, uses the rank order of the observed dissimilarities and looks for an optimal least-squares monotone increasing transformation in order to minimize the stress function (i.e., the badness-of-fit). It is often the preferred MDS method, as it does not make any assumptions regarding the relationship between the observed dissimilarity and the distance in space. As the two dimensions obtained via this method do not have a direct interpretation in our case, we have removed them from the graph in Fig 2 and have embedded instead two pieces of information from the original data matrix: the total “connectedness” of a single predictor (higher levels reflected by brighter color) and frequency of pairing (in an increasing manner from dotted to dashed to solid line).

Complimentary to MDS, we also conducted hierarchical cluster analysis employing a number of different methods (centroid, single, complete, average linkage, Ward’s error sum of squares). Determining the appropriate number of clusters was not straight-forward with any of these approaches – an issue most likely stemming again from the very nature of the dataset, containing a great many duplicate values and “disconnected” points (i.e., predictor variables that were very rarely, if at all, considered together with other predictors). Still, certain patterns of groupings were easily discernable across the different clustering procedures. The most apparent and persistent ones are incorporated into the MDS graph (Fig 2; grey contours) as well, though they should not be interpreted in any definitive way.

**References**

1. Bremberg SG. Mortality rates in OECD countries converged during the period 1990-2010. Scand J Public Healt. 2017;45(4):436-43.

2. Asandului M, Pintilescu C, Jemna D, Viorica D. Infant Mortality and the Socioeconomic Conditions in the Cee Countries after 1990. Transform Bus Econ. 2014;13(3c):555-65.

3. Khouri S, Cehlar M, Horansky K, Sandorova K. Expected Life Expectancy and Its Determinants in Selected European Countries. Transform Bus Econ. 2017;16(2b):638-55.

4. Reynolds MM, Avendano M. Social Policy Expenditures and Life Expectancy in High-Income Countries. Am J Prev Med. 2018;54(1):72-9.

5. Barthold D, Nandi A, Rodriguez JMM, Heymann J. Analyzing Whether Countries Are Equally Efficient at Improving Longevity for Men and Women. Am J Public Health. 2014;104(11):2163-9.

6. Budhdeo S, Watkins J, Atun R, Williams C, Zeltner T, Maruthappu M. Changes in government spending on healthcare and population mortality in the European union, 1995-2010: a cross-sectional ecological study. J Roy Soc Med. 2015;108(12):490-8.

7. Wubulihasimu P, Brouwer W, van Baal P. The Impact of Hospital Payment Schemes on Healthcare and Mortality: Evidence from Hospital Payment Reforms in OECD Countries. Health Econ. 2016;25(8):1005-19.

8. Lenhart O. The impact of minimum wages on population health: evidence from 24 OECD countries. Eur J Health Econ. 2017;18(8):1031-9.

9. Ferreira ER, Monteiro JD, Manso JRP. Are economic crises age and gender neutral? Evidence from European Union mortality data. Econ Anal Policy. 2018;60:69-77.

10. Reynolds MM. Health Care Public Sector Share and the US Life Expectancy Lag: A Country-level Longitudinal Study. Int J Health Serv. 2018;48(2):328-48.

11. Mackenbach JP. Cultural values and population health: a quantitative analysis of variations in cultural values, health behaviours and health outcomes among 42 European countries. Health Place. 2014;28:116-32.

12. Zare H, Gaskin DJ, Anderson G. Variations in life expectancy in Organization for Economic Co-operation and Development countries-1985-2010. Scand J Public Healt. 2015;43(8):786-95.

13. Park MB, Nam EW. National Level Social Determinants of Health and Outcomes: Longitudinal Analysis of 27 Industrialized Countries. Sage Open. 2019;9(2).

14. Korotayev A, Khaltourina D, Meshcherina K, Zamiatnina E. Distilled Spirits Overconsumption as the Most Important Factor of Excessive Adult Male Mortality in Europe. Alcohol Alcoholism. 2018;53(6):742-52.

15. Minagawa Y. Inequalities in Healthy Life Expectancy in Eastern Europe. Popul Dev Rev. 2013;39(4):649-71.

16. Borisova LV. Objective and Subjective Determinants of Self-Rated Health in Central and Eastern Europe: A Multilevel Approach. Cent Eur J Publ Heal. 2019;27(2):145-52.

17. Mackenbach JP, Hu YN, Looman CWN. Democratization and life expectancy in Europe, 1960-2008. Soc Sci Med. 2013;93:166-75.

18. Mackenbach JP, Looman CWN. Changing patterns of mortality in 25 European countries and their economic and political correlates, 1955-1989. Int J Public Health. 2013;58(6):811-23.

19. Bender KA, Economou A, Theodossiou I. The temporary and permanent effects of unemployment on mortality in Europe. Int Labour Rev. 2013;152(2):275-86.

20. Hu YN, van Lenthe FJ, Mackenbach JP. Income inequality, life expectancy and cause-specific mortality in 43 European countries, 1987-2008: a fixed effects study. Eur J Epidemiol. 2015;30(8):615-25.

21. Karyani AK, Kazemi Z, Shaahmadi F, Arefi Z, Meshkani Z. The Main Determinants of Under 5 Mortality Rate (U5MR) in OECD Countries: A Cross-Sectional Study. Int J Pediatr-Massha. 2015;3(1):421-7.

22. Bremberg SG. The rate of country-level improvements of the infant mortality rate is mainly determined by previous history. Eur J Public Health. 2016;26(4):597-601.

23. Filippidis FT, Laverty AA, Hone T, Been JV, Millett C. Association of Cigarette Price Differentials With Infant Mortality in 23 European Union Countries. Jama Pediatr. 2017;171(11):1100-6.

24. Tavares AI. Infant mortality in Europe, socio-economic determinants based on aggregate data. Appl Econ Lett. 2017;24(21):1588-96.

25. Bosakova L, Rosicova K, Bobakova DF, Rosic M, Dzurova D, Pikhart H, et al. Mortality in the Visegrad countries from the perspective of socioeconomic inequalities. Int J Public Health. 2019;64(3):365-76.

26. Tavares AI. eHealth, ICT and its relationship with self-reported health outcomes in the EU countries. Int J Med Inform. 2018;112:104-13.

27. Erdogan E, Ener M, Arica F. The Strategic Role of Infant Mortality in the Process of Economic Growth: An Application for High Income OECD Countries. Procd Soc Behv. 2013;99:19-25.

28. Mackenbach JP. Convergence and divergence of life expectancy in Europe: a centennial view. Eur J Epidemiol. 2013;28(3):229-40.

29. Mackenbach JP, Looman CWN. Life expectancy and national income in Europe, 1900-2008: an update of Preston's analysis. Int J Epidemiol. 2013;42(4):1100-10.

30. Baumbach A, Gulis G. Impact of financial crisis on selected health outcomes in Europe. Eur J Public Health. 2014;24(3):399-403.

31. Lopez-Casasnovas G, Soley-Bori M. The Socioeconomic Determinants of Health: Economic Growth and Health in the OECD Countries during the Last Three Decades. Int J Env Res Pub He. 2014;11(1):815-29.

32. Megyesiova S, Lieskovska V. The Relationship between the Health Status and Economic Situation in the Eu Member States. Int Multiddiscip Sci. 2014:1097-104.

33. Torre R, Myrskyla M. Income inequality and population health: An analysis of panel data for 21 developed countries, 1975-2006. Pop Stud-J Demog. 2014;68(1):1-13.

34. Iacob OC, Volintiru AM, Cristea A, Turcu E. Sustainable Position of European Countries Based on Life Expectancy at Birth and the Risk of Poverty. Sci Pap-Ser Manag Ec. 2015;15(4):117-24.

35. Safaei J. Distributional Orientation and Health Outcomes in OECD Countries. Int J Health Serv. 2015;45(4):601-21.

36. Xie RH, Gaudet L, Krewski D, Graham ID, Walker MC, Wen SW. Higher Cesarean Delivery Rates are Associated with Higher Infant Mortality Rates in Industrialized Countries. Birth-Iss Perinat C. 2015;42(1):62-9.

37. Shim J. Family leave policy and child mortality: Evidence from 19 OECD countries from 1969 to 2010. Int J Soc Welf. 2016;25(3):215-21.

38. Blazquez-Fernandez C, Cantarero-Prieto D, Pascual-Saez M. Health expenditure and socio-economic determinants of life expectancy in the OECD Asia/Pacific area countries. Appl Econ Lett. 2017;24(3):167-9.

39. Patton D, Costich JF, Lidstromer N. Paid Parental Leave Policies and Infant Mortality Rates in OECD Countries: Policy Implications for the United States. World Med Health Pol. 2017;9(1):6-23.

40. Richardson EA, Moon G, Pearce J, Shortt NK, Mitchell R. Multi-scalar influences on mortality change over time in 274 European cities. Soc Sci Med. 2017;179:45-51.

41. Tapia Granados JA, Ionides EL. Population health and the economy: Mortality and the Great Recession in Europe. Health Econ. 2017.

42. Kim JI, Kim G. Socio-ecological perspective of older age life expectancy: income, gender inequality, and financial crisis in Europe. Globalization Health. 2017;13.

43. Bartoll X, Mari-Dell'Olmo M. Patterns of life expectancy before and during economic recession, 2003-12: a European regions panel approach. Eur J Public Health. 2016;26(5):783-8.

44. Blazquez-Fernandez C, Cantarero-Prieto D, Pascual-Saez M. Does Rising Income Inequality Reduce Life Expectancy? New Evidence for 26 European Countries (1995-2014). Global Econ Rev. 2018;47(4):464-79.

45. Linden M, Ray D. Life expectancy effects of public and private health expenditures in OECD countries 1970-2012: Panel time series approach. Econ Anal Policy. 2017;56:101-13.

46. Pritchard C, Wallace MS. Comparing UK and Other Western Countries' Health Expenditure, Relative Poverty and Child Mortality: Are British Children Doubly Disadvantaged? Child Soc. 2015;29(5):462-72.

47. Pritchard C, Williams RJ, Wallace MS. Child mortality, health expenditure and poverty in the western nations 1979-2010: Are English-speaking countries' children disadvantaged? Childhood. 2015;22(1):138-44.

48. Marinacci C, Demaria M, Melis G, Borrell C, Corman D, Dell'Olmo MM, et al. The Role of Contextual Socioeconomic Circumstances and Neighborhood Poverty Segregation on Mortality in 4 European Cities. Int J Health Serv. 2017;47(4):636-54.

49. Ribeiro AI, Krainski ET, Carvalho MS, Launoy G, Pornet C, de Pina MD. Does community deprivation determine longevity after the age of 75? A cross-national analysis. Int J Public Health. 2018;63(4):469-79.

50. Laugesen M, Grace RC. Reduced tobacco consumption, improved diet and life expectancy for 1988-1998: analysis of New Zealand and OECD data. New Zeal Med J. 2017;130(1456):46-51.

51. Granados JAT, Ionides EL. Population health and the economy: Mortality and the Great Recession in Europe. Health Econ. 2017;26(12):E219-E35.

52. Kruskal JB, Wish M. Multidimensional Scaling. Beverly Hills, CA: Sage Publications; 1978.
