# Supplementary material for: Recent quantitative research on determinants of health in high income countries: A scoping review
Source: PLoS One. 2020 Sep 17;15(9):e0239031. doi: 10.1371/journal.pone.0239031 (PMC7498048; doi:10.1371/journal.pone.0239031)
Supplement: Attachment — Submitted filename: Response to Reviewers.docx [file pone.0239031.s005.docx]

Reviewer #3

*“English Language, syntax and punctuation needs substantial improvement. Don’t use long sentences.”*

- We carefully revised the manuscript, making edits throughout, and paying specific attention to cutting down long sentences. In addition, the manuscript was proofread by a fellow academic who is a native English speaker.

*“The 2nd paragraph of introduction has not been written in scientific language. Needs substantial change.”*

- The first two paragraphs have been revised as follows (lines 41-53):

“Identifying the key drivers of population health is a core subject in public health and health economics research. Between-country comparative research on the topic is challenging. In order to be relevant for policy, it requires disentangling different interrelated drivers of “good health”, each having different degrees of importance in different contexts.

“Good health” – physical and psychological, subjective and objective – can be defined and measured using a variety of approaches, depending on which aspect of health is the focus. A major distinction can be made between health measurements at the individual level or some aggregate level, such as a neighborhood, a region or a country. In view of this, a great diversity of specific research topics exists on the drivers of what constitutes individual or aggregate “good health”, including those focusing on health inequalities, the gender gap in longevity, and regional mortality and longevity differences.”

*“How this review and focusing on methods, can benefit the readership? Appraisal of methods could be a part of big review.”*

- The aim of this literature review was to examine the current state of research in the broad area of determinants of population health. We consider it beneficial (albeit disconcerting) to know that simplistic ways of approaching the problem are the preferred mode, and hope by pointing this out we will contribute towards a shift. Indeed, critically evaluating each statistical method we encountered in the selected papers was not feasible within the scope of our review, as outlined in the previous revision and the paper itself.

*“Page 5, line 73, please start a new paragraph.”*

- Implemented (line 70).

*“The added sentences from line 83-89 is suitable for discussion not introduction.”*

- In a previous round of the current review process, a reviewer asked us to justify our focus on high-income countries in the Introduction section. The referred lines were added in response to that request.

*“Please remove lines 89-91.”*

- Implemented.

*“In page 7, please put search strategy in a table, not in the text.”*

- Implemented. The table has been removed from the main text and included as an appendix, as per Reviewer #4’s suggestion.

*“Who developed and performed the search strategy? Was a librarian consulted? What is PICO?”*

- The search strategy was developed iteratively between the two authors, benefiting from our research centre’s vast previous experience performing systematic literature reviews. A librarian was not consulted. The search was performed by the first author.

The PICO (population, intervention, comparison, outcome) framework for formulating a research question is not applicable to our review and applying it is also not required for a scoping review(1).

*“Line 134, please provide Kappa statistics, and intra and inter rater agreements”*

- The percentage agreement we have provided is, indeed, an inter-rater reliability measure. In the context of a literature review paper, we find citing that percentage and the number of cases where discussion between the two authors was needed more informative than Kappa statistics.

*“Line 137, how do you define “aggregate level factor”?”*

- The definition was provided in lines 49-50 in the Introduction section: “or some aggregate, such as a neighborhood, a region or a country”; as well as in line 91 in the Methods section: “aggregate level (region/city/country) determinant”. In other words, any factor that was measured on a region-, city-, country-level, etc. For the sake of maximum clarity, the text referred to was changed as follows (lines 107-111): “(a) the paper was a full published report of an original empirical study investigating the impact of at least one aggregate level (city/region/country) factor on at least one health indicator (or self-reported health) of the general population (the only admissible “sub-populations” were those based on gender and/or age)”.

*“Line 151, the single person used for date extraction does not seem reliable. It is suggested that at least 2 persons should independently extract data, or the single data extractor be calibrated previously.”*

- Data extraction in this particular case was straight-forward and the labor of both authors with regards to this part of the review was not considered necessary. Any uncertainty was discussed and resolved between the two authors.

*“Page 8, please include and describe data analysis method utilized in your study”*

- We consider all of the following components to be part of our data analysis method: search strategy, eligibility criteria, data extraction and evidence synthesis. We have described those in the respective sub-sections of the Methods section (p.6-7).

*“line 552-554, please modify this statement. This is broad and you have only focused on methods.”*

- We would like to point out that we investigated types of health outcomes used, regions, countries and populations, data types and sources, health determinants, statistical methodology, and results (as reflected in the different sub-sections of our Results section), thus going beyond the strict definition of “methods”.

*“Considering the focus of the review on methods, the discussion needs to be richer and critically appraise the findings with regards to methods (not studies, as justified in line 596). How the findings of these review can be helpful? Etc…”*

- If we may re-print here a response to a previous PLOS ONE reviewer’s similar comment earlier in the referee process for this paper: “We found it impractical to attempt to critically discuss specific statistical approaches when so many of them were encountered. With the aim of providing an overview of how research on the very broad topic of determinants of population health has been conducted, we chose to stay descriptive, nevertheless suggesting on numerous ocassions that methods used were not necessarily optimal.” And to reiterate, our general conclusion that methodological shortcomings may be holding research in this field back is hoped to serve as guidance for researchers to advance the field in future research.

*“I believe some explanations provided for past reviews can be implemented into the text for the convenient of reads too. Please do so when possible.”*

- Referring to past reviews is conventionally done in the Introduction section to provide context for the current review. If appropriate, they could again be cited in the Discussion section. However, due to the differences in scope and focus between our review and all others we are aware of, no further mention of past reviews was deemed justified or necessary.

Reviewer #4

*“I suggest that the search string be added to the supplementary material of the manuscript.”*

- The search strategy has been re-formatted as a table as per Reviewer #3’s suggestion and provided in an appendix.

*“The footnotes may be show at the appendix file where it is located the figure 2. At time, please, review figure 2 and add the title.”*

- We refer to PLOS ONE’s submission guidelines with regards to figure captions: “Figure captions must be inserted in the text of the manuscript, immediately following the paragraph in which the figure is first cited (read order). Do not include captions as part of the figure files themselves or submit them in a separate document. … The caption may also include a legend as needed.” (<https://journals.plos.org/plosone/s/submission-guidelines#loc-supporting-information>)

*“If possible, try to reduce the number of pages and focus on the primary objective of the manuscript.”*

- We have critically revised and reduced the text also in line with the other referee comments made, emphasizing the primary objectives.

REFERENCES

1.	Tricco AC, Lillie E, Zarin W, O'Brien KK, Colquhoun H, Levac D, et al. PRISMA Extension for Scoping Reviews (PRISMA-ScR): Checklist and Explanation. Ann Intern Med. 2018;169(7):467-+.
