# Supplementary material for: Recent quantitative research on determinants of health in high income countries: A scoping review
Source: PLoS One. 2020 Sep 17;15(9):e0239031. doi: 10.1371/journal.pone.0239031 (PMC7498048; doi:10.1371/journal.pone.0239031)
Supplement: Attachment — Submitted filename: Response to Reviewers.docx [file pone.0239031.s006.docx]

Reviewer #3

*“In abstract background, provide aim of the study.”*

- We have edited the abstract’s “Background” section to include the aim (lines 10-13): “**Background:** Identifying determinants of health and understanding their role in health production constitutes an important research theme. We aimed to document the state of recent multi-country research on this theme in the literature.”

*“In the abstract methods section, please give more details about methods and move the results part “Sixty studies that performed cross-national statistical analyses aiming to evaluate the impact of one or more aggregate level determinants on one or more general population health outcomes in high-income countries were selected” to results.”*

- The sentence in question was modified in the “Methods” part as follows (lines 15-18): “We searched for studies that performed cross-national statistical analyses aiming to evaluate the impact of one or more aggregate level determinants on one or more general population health outcomes in high-income countries.” The sentence “Sixty studies were selected, out of an original yield of 3686.” was placed in the beginning of the “Results” section (line 21).

*“In abstract result, please modify last sentence as “effects fluctuated between statistically significant and not significant, and between beneficial to health and detrimental to health”.”*

- The sentence has been changed to (lines 30-33): “Across a multitude of model specifications, different country samples, and varying time periods, effects fluctuated between statistically significant and not significant, and between beneficial and detrimental to health.”

*“Merge the answer to comment “How this review and focusing on methods, can benefit the readership? Appraisal of methods could be a part of big review.” To the first paragraph of discussion.”*

- The points made in each of the sentences suggested for inclusion are already present in the manuscript, as follows:

**“The aim of this literature review was to examine the current state of research in the broad area of determinants of population health.”**

A more detailed version of this is presented in the opening sentence of the “Discussion” section (lines 523-525): “The purpose of this scoping review was to examine recent quantitative work on the topic of multi-country analyses of determinants of population health in high-income countries.” as well as in the concluding remarks (lines 575-577): “Our aim was to investigate the current state of research on the very broad and general topic of population health, specifically, the way it has been examined in a multi-country context.”

**“We consider it beneficial (albeit disconcerting) to know that simplistic ways of approaching the problem are the preferred mode, and hope by pointing this out we will contribute towards a shift.”**

This message appears in the paper on three different occasions: in the “Conclusions” of the abstract (lines 34-39): “We conclude that efforts to understand the underlying mechanisms of population health are far from settled, and the present state of research on the topic leaves much to be desired. It is essential that future research considers multiple factors simultaneously and takes advantage of more sophisticated methodology with regards to quantifying health as well as analyzing determinants’ influence.”; much more elaborately in the 4^th^ paragraph of the “Discussion” section dealing with health determinants and analytical approaches (lines 546-558): “Nevertheless, the graph is helpful in exemplifying how many effects continue to be studied in a very limited context, if any. Since in reality no factor acts in isolation, this oversimplification practice threatens to render the whole exercise meaningless from the outset. The importance of multivariate analysis cannot be stressed enough. While there is no “best method” to be recommended and appropriate techniques vary according to the specifics of the research question and the characteristics of the data at hand, in the future, in addition to abandoning simplistic univariate approaches, we hope to see a shift from the currently dominating fixed effects to the more flexible random/mixed effects models, as well as wider application of more sophisticated methods, such as principle component regression, partial least squares, covariance structure models (e.g., structural equations), canonical correlations, time-series, and generalized estimating equations.”; and finally, as a “take-home message” in the last lines 577-583: “We learned that data treatment and analytical approach were, in the majority of these recent studies, ill-equipped or insufficiently transparent to provide clarity regarding the underlying mechanisms of population health in high-income countries. Whether due to methodological shortcomings or the inherent complexity of the topic, research so far fails to provide any definitive answers. It is our sincere belief that with the application of more advanced analytical techniques this continuous quest could come to fruition sooner.”

**“Indeed, critically evaluating each statistical method we encountered in the selected papers was not feasible within the scope of our review, as outlined in the previous revision and the paper itself.”**

This was written in direct response to the reviewer’s statement: “*Appraisal of methods could be a part of big review.”* We never promised the reader such an appraisal (as we believe it lies outside the scope of this review) and thus did not find it necessary to explicitly “defend” ourselves in the manuscript why we did not do it. Instead, we chose to suggest on numerous occasions that methods encountered in the review were not necessarily optimal (e.g., line 411: “listed in increasing level of sophistication”; line 416: “Among the more advanced statistical methods”; lines 433-434: “some other simplistic approaches to longitudinal data analysis were found”; line 438: “Moving further in the direction of more sensible longitudinal data usage”), at the same time saying that “there is no “best method” to be recommended and appropriate techniques vary according to the specifics of the research question and the characteristics of the data at hand” (lines 550-552), supplemented by some statistical textbook references (line 553).

*“Mention the answer to “Who developed and performed the search strategy? Was a librarian consulted? What is PICO?” in the methods section.”*

- Implemented (lines 97-102): “The search strategy was developed iteratively between the two authors and is presented in S1 Appendix. The search was performed by VV in PubMed and Web of Science on the 16^th^ of July, 2019, without any language restrictions, and with a start date set to the 1^st^ of January, 2013, as we were interested in the latest developments in this area of research.”

*“The reviewer intention from comment “I believe some explanations provided for past reviews can be implemented into the text for the convenient of reads too. Please do so when possible” was to ask the authors to include their explanations for reviewers in the past round of review into the manuscript. The authors have provided some good explanations in the past round of review which can benefit readers too. So please add your explanations to reviewers in the past round of review to the text when possible.”*

- After reviewing our earlier responses, we do not think inserting those would benefit the text, as we want to avoid differently phrased repetitions in the paper. Additionally, if the review is accepted for publication, as per PLOS ONE policy, we will also be given the option of making the peer review history open, thus giving readers access to this text as well.
